# iTRAQ-Based Proteomic Analysis of Sublethally Injured *Escherichia coli* O157:H7 Cells Induced by High Pressure Carbon Dioxide

**DOI:** 10.3389/fmicb.2017.02544

**Published:** 2017-12-18

**Authors:** Xiufang Bi, Yongtao Wang, Xiaosong Hu, Xiaojun Liao

**Affiliations:** ^1^Beijing Advanced Innovation Center for Food Nutrition and Human Health, College of Food Science and Nutritional Engineering, China Agricultural University, Beijing, China; ^2^Key Lab of Fruit and Vegetable Processing, Ministry of Agriculture, Beijing, China; ^3^Sichuan Key Laboratory of Food Bio-technology, College of Food and Bioengineering, Xihua University, Chengdu, China

**Keywords:** *Escherichia coli* O157:H7, sublethal injury, high pressure carbon dioxide, proteome, stress response

## Abstract

High pressure carbon dioxide (HPCD) could cause sublethally injured cells (SICs), which may cause food poisoning and spoilage during food storage and limit its application. Therefore, the formation of SICs of *Escherichia coli* O157:H7 was investigated by isobaric tag for relative and absolute quantification (iTRAQ) proteomic methods in this study for better controlling the SICs induced by HPCD. A total of 2,446 proteins was identified by iTRAQ, of which 93 and 29 were significantly differentially expressed in the SICs compared with live control cells (CK_L_) and dead control cells (CK_D_), respectively. Among the 93 differentially expressed proteins (DEP) in the SICs compared with CK_L_, 65 proteins showed down-regulation and 28 showed up-regulation. According to the comprehensive proteome coverage analysis, the SICs survived under HPCD by reducing carbohydrate decomposing, lipid transport and metabolism, amino acid transport and metabolism, transcription and translation, DNA replication and repair. Besides, the SICs showed stress response, DNA damage response and an increased carbohydrate transport, peptidoglycan synthesis and disulfide bond formation to HPCD. Among the 29 DEP in the SICs compared with CK_D_, 12 proteins showed down-regulation and 17 showed up-regulation. According to the comprehensive proteome coverage analysis, the SICs survived under HPCD by accumulation of cell protective agents like carbohydrates and amino acids, and decreasing transcription and translation activities. Results showed that the formation of the SICs with low metabolic activity and high survival ability was a survival strategy for *E. coli* O157:H7 against HPCD.

## Introduction

*Escherichia coli* O157:H7 is a type of enterohemorrhagic, Shiga toxin producing *E. coli* (EHEC) first indentified in 1982 (Riley et al., 1983). This pathogen could cause foodborne outbreaks worldwide, e.g., foodborne outbreaks, including 4,928 illnesses, 1,272 hospitalizations, and 33 deaths, were identified to be caused by *E. coli* O157:H7 in the United States from 2003 to 2012 (Heiman et al., 2015). The majority of outbreaks caused by *E. coli* O157:H7 have been linked to the consumption of contaminated foods such as beef, leafy vegetables, and drinking water (Marder et al., 2014; Heiman et al., 2015). It has been reported the infective dose of *E. coli* O157:H7 was low and may be as few as 10 cells (Phillips, 1999). In the efforts to improve food safety by inactivating *E. coli* O157:H7 cells, thermal pasteurization techniques are widely used. However, nutritive compounds, color, texture, taste and aroma could be destroyed, especially for fruits and vegetables products. Therefore, non-thermal pasteurization techniques, which aim to achieve similar microbial with minimal impact on food quality, have gained much attention (Garcia-Gonzalez et al., 2007).

High pressure carbon dioxide (HPCD), a novel non-thermal technology, can effectively inactivate *E. coli* in carrot juice (Bi et al., 2014) and apple juice (Liao et al., 2007). The bactericidal effects, kinetics and mechanisms of HPCD-induced inactivation have been mostly investigated (Liao et al., 2007, 2011). In recent years, researchers found that some sublethally injured cells (SICs) may survive after HPCD despite having sustained membrane damage (Yuk and Geveke, 2011; Bi et al., 2015). Bi et al. (2015) found HPCD caused sublethal injury to *E. coli* O157:H7 cells, which could not recover in the selective medium, leading to an overestimation of lethality of HPCD processing. However, the SICs could recover during storage under favorable conditions, posing a potential health risk for HPCD-treated products (Garcia-Gonzalez et al., 2007). In order to understand and control the SICs induced by HPCD, it is very important to understand how SICs were formed during HPCD processing.

Until now, studies on the SICs caused by non-thermal pasteurization techniques are mainly focused on its induction, recovery and physiological properties (Sokołowska et al., 2014; Bi et al., 2015; Wang et al., 2015; Li et al., 2017). Only a few studies were carried out on molecular characteristics of the SICs which could help reveal its formation mechanisms (Ulmer et al., 2002; Molina-Höppner et al., 2004; Kilimann et al., 2006; Zhao et al., 2014a,b). However, these studies investigated the relationship between formation of the SICs and a few membrane-related enzymes (e.g., Ca^2+^-ATPase, LmrP, HorA), which can partly reveal the formation mechanisms of the SICs induced by high pressure and pulse electric fields (PEF).

Quantitative proteomics, firstly proposed by Wilkins and Williams in 1994, has been used in the mechanistic study of cell responses to stress (Jin et al., 2017). Using two-dimensional electrophoresis (2-DE) proteomic analysis, Rivas et al. (2013) found the protein profile of the SICs of *E. coli* DH5α induced by PEF was significantly different from the untreated cells, and the differentially expressed proteins (DEP) were mainly some structural and metabolic proteins (ompA, gmhA, ClpA, RS6, Dut, FtnA, TufB, ftsH, putA, atpA, and sdhA). By analyzing the function of these DEP, a better understanding of the formation mechanisms of the SICs induced by PEF was achieved (Rivas et al., 2013). To our knowledge, no study has been carried out on the proteomic profiles of the SICs induced by HPCD so far. The isobaric tags for relative and absolute quantification (iTRAQ) combined with multidimensional liquid chromatography and tandem MS analysis is one of most powerful methodologies in quantitative proteomics (Yang et al., 2016). In addition, it allows for simultaneous relative quantification of up to eight samples within a single run, which could ensure the confidence of comparison. Therefore, iTRAQ-based quantitative proteomics analysis was applied to investigate the proteomic profiles in the SICs of *E. coli* O157:H7 induced by HPCD in this study.

## Materials and methods

### Chemicals

Tetraethylammonium bromide (TEAB) and iTRAQ reagent were purchased from Applied Biosystems (Milan, Italy). Tryptic soy broth (TSB) was purchased from Beijing Aoboxing Biological Technology Co. Ltd (Beijing, China). Bradford protein assay kit was purchased from Thermo Fisher Scientific (Massachusetts, USA). Trypsin Gold was purchased from Promega Corporation (Wisconsin, USA). Acetonitrile (ACN) and formic acid (FA) of high-performance liquid chromatography (HPLC) grade were purchased from Honeywell Burdick & Jackson (SK Chemicals, Seoul, Korea). Other chemicals were obtained from Beijing Chemicals Co. (Beijing, China).

### Bacterial strain and induction of the SICs by HPCD treatment

*E. coli* O157:H7 NCTC 12900 was obtained from the National Culture Type Collection (Colindale, London, United Kingdom). *E. coli* cells grown in TSB at 37°C for 3 h (intermediate exponential phase) were centrifuged at 7669.4 *g* for 10 min at 4°C (CF16RXII, HITACHI, Japan). The harvested cells were then washed and resuspended in sterile phosphate-buffered saline (PBS: 10 mM potassium phosphate buffer, 8.4 g/L NaCl, pH 7.00) (Beijing Chemical Works, Beijing, China). In accordance with the procedure described by Bi et al. (2015), sublethally injured *E. coli* cells (≈10^7^ CFU/mL) were generated by HPCD at 5 MPa and 37°C for 15 min. HPCD treatment was performed with a batch HPCD system (Liao et al., 2007). Twenty milliliter of *E. coli* cells was transferred to a 50 mL sterile glass tube and the tube was covered with a plastic film (Beijing Lanyi chemical products Co. Ltd., Beijing, China) with a 0.22 μm membrane filter in the center for aeration and preventing microbial contamination. As the pressure vessel of the HPCD system reached the experimental temperature, the sample tubes were placed in the pressure vessel. The vessel was pressurized by the plunger pump to required pressure and held for the required treatment time. Then, the depressurization was performed by opening the pressure relief valve of CO_2_. The compression time was in the range of 40–60 s and the depressurization time was 30–60 s. After HPCD treatment, the percentages of intact cells, dead cells, and SICs in suspension were 32.7, 21.4, and 45.9%, respectively (Bi et al., 2015). Therefore, both intact cells and dead cells were used as controls in the following study. Untreated *E. coli* cells at intermediate exponential phase served as control of intact live cells (CK_L_, ≈10^8^ CFU/mL), and cells treated by HPCD at 5 MPa and 37°C for 40 min was used as control of dead cells (CK_D_, ≈10^7^ CFU/mL).

### Protein preparation

Ten replicates of untreated and HPCD-treated *E. coli* cells were collected and mixed evenly, separately. Then the proteins were extracted according to Liao et al. (2011) with some modifications. The cells were washed with sterile phosphate buffered saline (PBS, pH 7.0), and ground into powder in liquid nitrogen, extracted with Lysis buffer (7 M Urea, 2 M Thiourea, 4% CHAPS, 40 mM Tris-HCl, pH 8.5) containing 1 mM phenylmethylsulfonyl fluoride (PMSF) and 2 mM EDTA (final concentration). After 5 min, 10 mM dithiothreitol (DTT, final concentration) was added to the samples. The suspension was sonicated at 200 W for 15 min (JY92-II, Ningbo Xinzhi biological Polytron Technologies Inc., Ningbo, China) and then centrifuged at 25,000 g and 4°C for 20 min (CF16RXII, Hitachi, Ltd., Tokyo, Janpan). The supernatant was mixed well with 5 × volume of chilled acetone containing 10% (v/v) trichloroacetic acid (TCA) and incubated at −20°C for 2 h. After centrifugation at 16,000 g and 4°C for 20 min, the supernatant was discarded. The precipitate was dissolved in Lysis buffer (7 M urea, 2 M thiourea, 4% NP40, 20 mM Tris-HCl, pH 8.0–8.5) containing 1 mM PMSF and 2 mM EDTA (final concentration). After 5 min, 10 mM DTT (final concentration) was added to the samples. The suspension was sonicated at 200 W for 15 min and centrifuged at 25,000 g and 4°C for 20 min. The supernatant was collected.

To reduce disulfide bonds in proteins of the supernatant, 10 mM DTT (final concentration) was added and incubated at 56°C for 1 h. Subsequently, 55 mM iodoacetamide (IAM, final concentration) was added to block the cysteines, incubated for 45 min in the darkroom. The supernatant was mixed well with 55 × volume of chilled acetone for 2 h at −20°C to precipitate proteins. After centrifugation at 25,000 g and 4°C for 20 min, the supernatant was discarded, and the pellet was air-dried for 5 min, dissolved in 200 μL 0.5 M TEAB and sonicated at 200 W for 15 min. Finally, samples were centrifuged at 25,000 g and 4°C for 20 min. The supernatant was collected and the proteins were quantified, using Bradford protein assay kit. The proteins in the supernatant were kept at −80°C for further analysis.

### iTRAQ labeling and strong cation exchange (SCX) chromatography

According to the iTRAQ protocol provided by Applied Biosystems (Milan, Italy), total protein (100 μg) was taken out of each sample solution and then the protein was digested with Trypsin Gold with the ratio of protein: trypsin = 30:1 at 37°C for 16 h. After trypsin digestion, peptides were dried by vacuum centrifugation. Peptides were reconstituted in 0.5 M TEAB and processed according to the manufacture's protocol for 8-plex iTRAQ reagent. Briefly, one unit of iTRAQ reagent was thawed and reconstituted in 24 μL isopropanol. Samples were labeled with the iTRAQ tags as follow: sample SICs (115 tag), sample CK_L_ (113 tag), sample CK_D_ (119 tag). Samples were tested in duplicates in a single run. The peptides were labeled with the isobaric tags, incubated at room temperature for 2 h. The labeled peptide mixtures were then pooled and dried by vacuum centrifugation.

SCX chromatography was performed as described by Pandhal et al. (2011) with a LC-20AB HPLC Pump system (Shimadzu, Kyoto, Japan). The iTRAQ labeled peptide mixtures were reconstituted with 4 mL buffer A (25 mM NaH_2_PO_4_ in 25% ACN, pH 2.7) and loaded onto a 4.6 × 250 mm Ultremex SCX column containing 5-μm particles (Phenomenex, California, USA). The peptides were eluted at a flow rate of 1 mL/min with a gradient of buffer A for 10 min, 5–60% buffer B (25 mM NaH_2_PO_4_, 1 M KCl in 25% ACN, pH 2.7) for 27 min, 60–100% buffer B for 1 min. The system was then maintained at 100% buffer B for 2 min before equilibrating with buffer A for 10 min prior to the next injection. Elution was monitored by measuring the absorbance at 214 nm, and fractions were collected every 1 min. The eluted peptides were pooled into 12 fractions, desalted with a Strata X C18 column (Phenomenex, California, USA) and vacuum-dried.

### LC-ESI-MS/MS analysis

According to Zhao et al. (2016), each fraction was resuspended in buffer A (5% ACN, 0.1% FA) and centrifuged at 20,000 g for 10 min, the final concentration of peptide was about 0.5 μg/μL on average. Ten microliters supernatant was loaded on a LC-20AD nano-HPLC (Shimadzu, Kyoto, Japan) by the auto-sampler onto a 2 cm C18 trap column. Then, the peptides were eluted onto a 10 cm analytical C18 column (inner diameter 75 μm) packed in-house. The samples were loaded at 8 μL/min for 4 min, then the 35 min gradient was run at 300 nL/min starting from 2 to 35% B (95% ACN, 0.1% FA), followed by 5 min linear gradient to 60%, then, followed by 2 min linear gradient to 80%, and maintenance at 80% B for 4 min, and finally return to 5% in 1 min. Data acquisition was performed with a TripleTOF 5600 System (AB Sciex Pte. Ltd., Ontario, Canada) fitted with a Nanospray III source (AB Sciex Pte. Ltd., Ontario, Canada) and a pulled quartz tip as the emitter (New Objectives, Massachusetts, USA). Data was acquired using an ion spray voltage of 2.5 kV, curtain gas of 30 psi, nebulizer gas of 15 psi, and an interface heater temperature of 150°C. The MS was operated with a reverse phase (≥30,000 FWHM) for TOF MS scans. For IDA, survey scans were acquired in 250 ms and as many as 30 product ion scans were collected if exceeding a threshold of 120 counts per second (counts/s) and with a 2+ to 5+ charge-state. Total cycle time was fixed to 3.3 s. Q2 transmission window was 100 Da for 100%. Four time bins were summed for each scan at a pulser frequency value of 11 kHz through monitoring of the 40 GHz multichannel TDC detector with four-anode channel detect ion. A sweeping collision energy setting of 35 ± 5 eV coupled with iTRAQ adjust rolling collision energy was applied to all precursor ions for collision-induced dissociation. Dynamic exclusion was set for 1/2 of peak width (15 s), and then the precursor was refreshed off the exclusion list.

### Data analysis

Data was analyzed by method proposed by Zhao et al. (2016). Raw data files acquired from the Orbitrap were converted into MGF files using Proteome Discoverer 1.2 (PD 1.2, Thermo), and the MGF files were searched. Proteins identification was performed by using Mascot search engine (Matrix Science, London, UK; version 2.3.02) against the protein translation database containing *E. coli* O157:H7 sequences. For protein identification, a mass tolerance of 0.05 Da (ppm) was permitted for intact peptide masses and 0.1 Da for fragmented ions, with allowance for one missed cleavages in the trypsin digests, Gln->pyro-Glu (N-term Q), Oxidation (M), iTRAQ8plex (Y) as the potential variable modifications, and Carbamidomethyl (C), iTRAQ8plex (N-term), iTRAQ8plex (K) as fixed modifications. The charge states of peptides were set to +2 and +3. Specifically, an automatic decoy database search was performed in Mascot by choosing the decoy checkbox in which a random sequence of database is generated and tested for raw spectra as well as the real database. To reduce the probability of false peptide identification, only peptides with significance scores (≥20) at the 99% confidence interval by a Mascot probability analysis greater than “identity” were counted as identified. Each confident protein identification involved at least one unique peptide. The GO database (http://www.geneontology.org) and the COG database (http://www.ncbi.nlm.nih.gov/COG/) were used to classify and group the identified proteins. For protein quantitation, it was required that a protein contains at least two unique peptides. The quantitative protein ratios were weighted and normalized by the median ratio in Mascot. For data analysis, *t*-test was used to determine statistical differences, and protein quantification data with relative expression of >1.5 and <0.67 and *P* < 0.05 was chosen to ensure up- and down regulation authenticity. Functional annotations of the DEP were conducted against Uniprot database (http://www.uniprot.org).

## Results

### Identification of proteins

**Figure 1:**
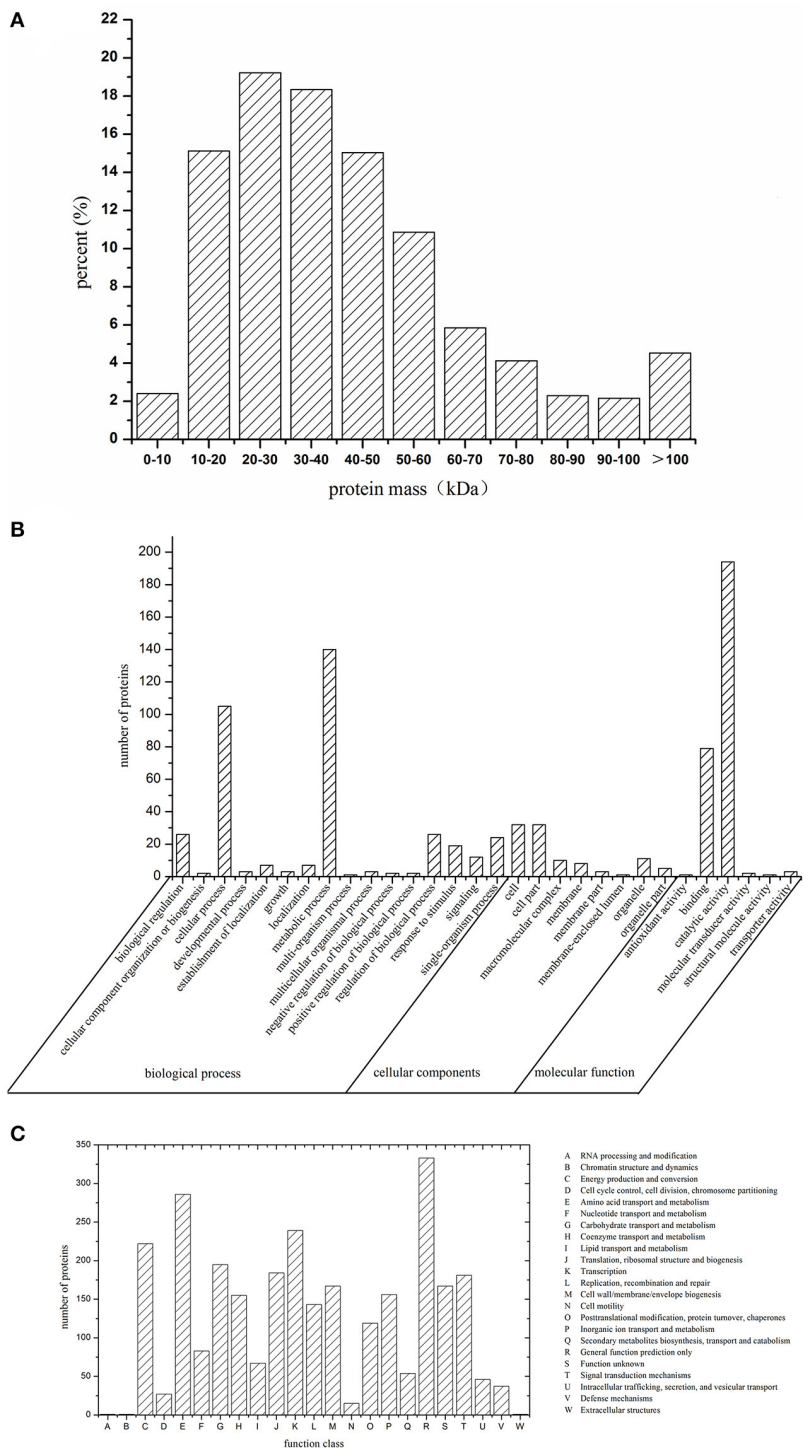
Molecular mass **(A)**, GO classification **(B)**, and COG classification **(C)** of identified proteins of sublethally injured cells, live cells, and dead cells.

The iTRAQ method was performed to identify the proteins that were differentially expressed between the SICs and CK_L_, or SICs and CK_D_. A total of 17,060 unique peptides associated with 2,446 proteins were identified (Table S1). The molecular masses of these proteins were mainly in the range of 10–60 kDa (Figure 1A), and functional classification of the total identified proteins was performed by GO (Figure 1B) and COG analysis (Figure 1C). The 382 proteins were classified into 16 functional categories according to the biological process listed on the GO database. Among these proteins, the top five categories were involved in metabolic process (36.65%), cellular process (27.49%), biological regulation (6.81%), regulation of biological process (6.81%) and single-organism process (6.82%). The 102 proteins were classified into eight classes according to the cell component in GO database. These proteins were mainly located at cell (31.37%), cell part (31.37%), organelle (10.78%), macromolecular complex (9.80%), and membrane (7.84%). The 280 proteins were classified into 6 categories according to molecular function in COG database. These proteins mainly involved in catalytic activity (69.28%) and binding (28.21%). The 2,446 proteins were classified into 23 COG function categories (Figure 1C). Among these proteins, the top 10 categories were involved in general function prediction only (11.57%), amino acid transport and metabolism (9.93%), transcription (8.30%), energy production and conversion (7.71%), carbohydrate transport and metabolism (6.77%), translation, ribosomal structure and biogenesis (6.39%), signal transduction mechanisms (6.29%), cell wall/membrane/envelope biogenesis (5.80%), inorganic ion transport and metabolism (5.42%).

### Functional classification of differentially expressed proteins (DEP)

**Figure 2:**
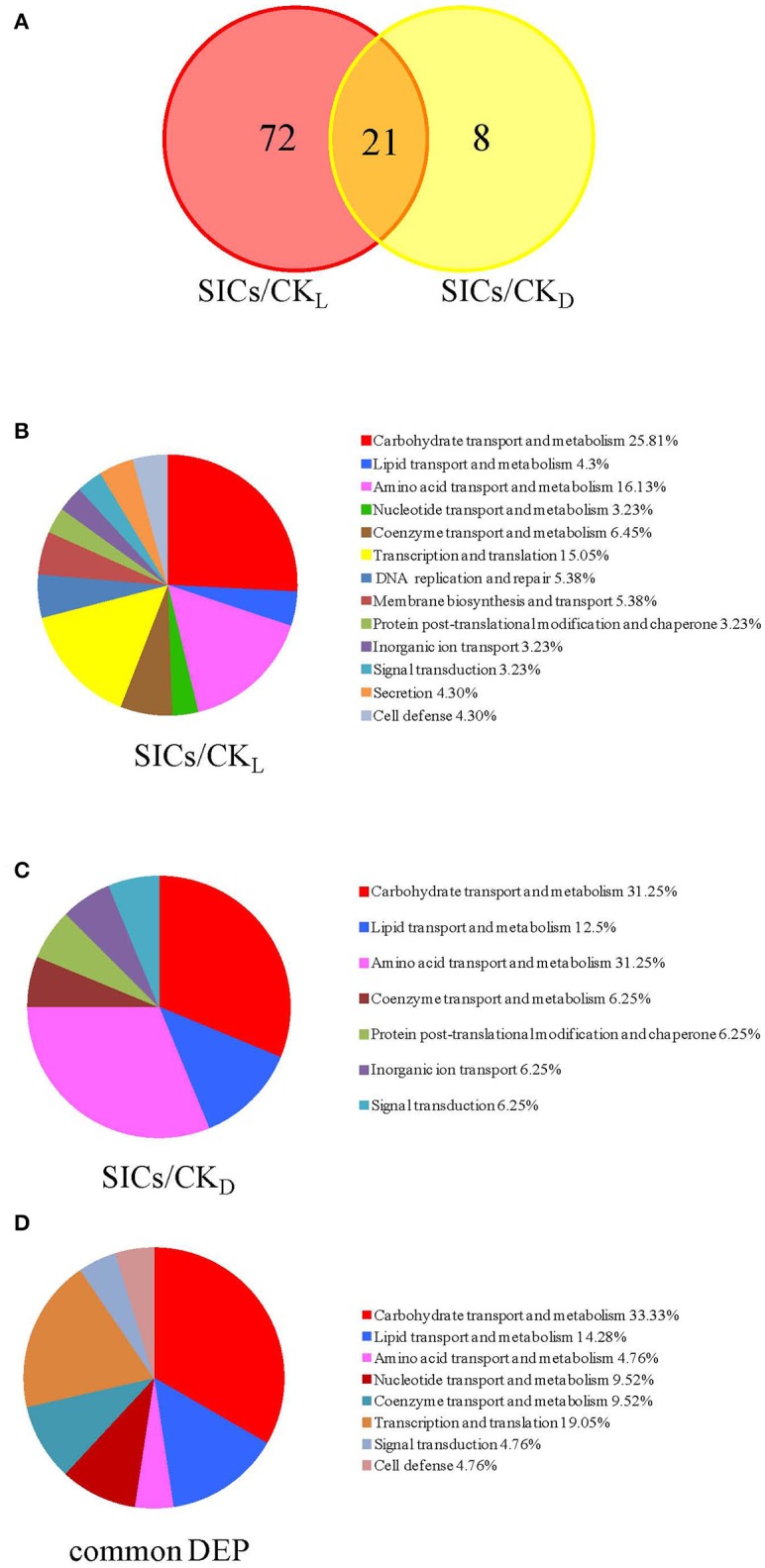
Venn diagrams of the differentially expressed proteins **(A)** and functional classifications of these proteins in sublethally injured cells compared with live cells **(B)**, dead cells **(C)**, and proteins in common **(D)**. DEP means differentially expressed proteins; SICs means sublethally injured cells; CK_L_ means control of live cells; CK_D_ means control of dead cells.

**Figure 3:**
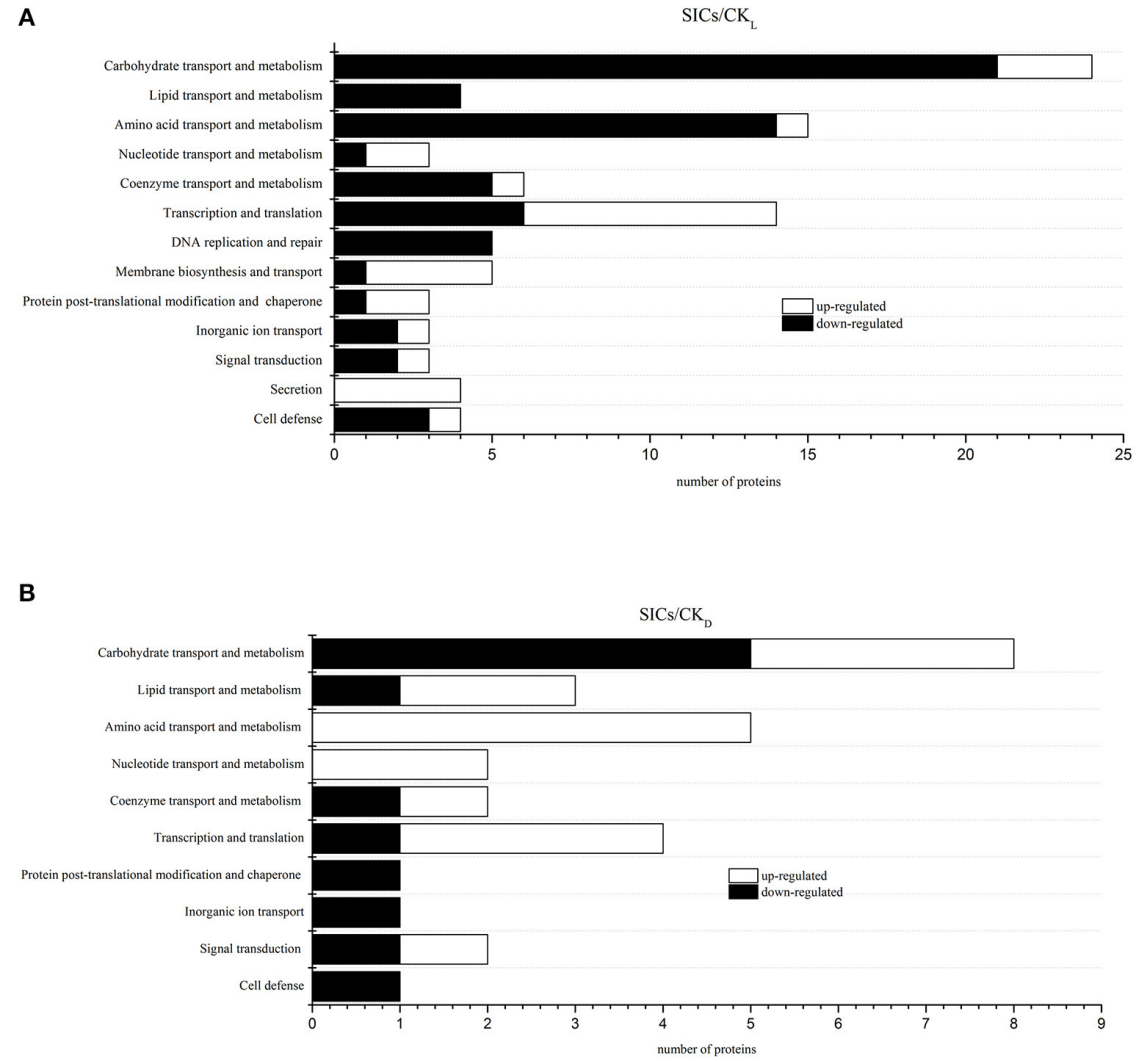
Classification of differentially expressed proteins in sublethally injured cells compared with live cells **(A)** and dead cells **(B)**. SICs means sublethally injured cells; CK_L_ means control of live cells; CK_D_ means control of dead cells.

In our study, the iTRAQ reporter ratios of 1.5- and 0.67-fold (*p* < 0.05) were set as the cut-off value for protein changes. A total of 93 and 29 DEP was found in the SICs compared with CK_L_ and CK_D_ cells, respectively, and 21 DEP in all the samples (Figure 2A). Detailed information about every DEP was listed in Table 1. Among the 93 DEP in the SICs compared with CK_L_, 65 proteins showed down-regulation and 28 showed up-regulation (Figure 3A), and the protein functions were classified into 13 categories according to functional information provided by Uniprot database (Figure 2B). The main functional categories were carbohydrate transport and metabolism (25.81%), amino acid transport and metabolism (16.13%), transcription and translation (15.05%), coenzyme transport and metabolism (6.45%), DNA replication and repair (5.38%) and membrane biosynthesis and transport (5.38%) (Figure 2B). Among the 29 DEP in the SICs compared with CK_D_, 12 proteins showed down-regulation and 17 proteins showed up-regulation (Figure 3B), and the protein functions were classified into 10 categories according to functional information provided by Uniprot database (Figure 2C). The main functions categories were carbohydrate transport and metabolism (27.58%), amino acid transport and metabolism (17.24%), transcription and translation (13.79%), and lipid transport and metabolism (10.34%) (Figure 2C). The 21 common DEP found in both SICs/CK_L_ and SICs/CK_D_ were classified into 8 categories according to functional information provided by Uniprot database, and the main categories were carbohydrate transport and metabolism (33.33%), lipid transport and metabolism (14.28%), and transcription and translation (19.05%) (Figure 2D).

**Table 1:** Differentially expressed proteins in sublethally injured cells induced by high pressure carbon dioxide treatment.

**NCBI Accession No**.	**Description**	**Fold change**	
		**SICs/CK_L_**	**SICs/CK_D_**
**CARBOHYDRATE TRANSPORT AND METABOLISM**			
gb|EZQ45808.1|	Fructose-bisphosphate aldolase	0.563	–
gb|EZQ54756.1|	Fructose-6-phosphate aldolase	0.604	–
gb|EZQ52788.1|	Succinate dehydrogenase	0.497	0.503
gb|ELW07811.1|	Fumarate hydratase class I	0.469	–
gb|EZQ50551.1|	Isocitrate lyase	0.54	2.924
gb|EZQ50401.1|	Fumarate reductase	0.558	–
gb|EZQ53317.1|	Formate dehydrogenase	0.299	–
gb|EZQ55021.1|	Nitrite reductase	0.387	–
gb|ELW31130.1|	Nitrate reductase molybdenum cofactor assembly chaperone	0.454	–
gb|EZQ54830.1|	Dimethyl sulfoxide reductase	0.479	–
gb|EZQ48937.1|	Beta-D-galactosidase	0.463	–
gb|EZQ54070.1|	PTS sugar transporter	1.55	1.783
gb|EZQ52698.1|	Alpha-dehydro-beta-deoxy-D-glucarate aldolase	0.348	0.59
gb|EZQ52697.1|	Galactonate transporter	0.263	0.478
gb|ELV67595.1|	Glucarate dehydratase	0.486	0.618
gb|EZQ54551.1|	Glycerol-3-phosphate dehydrogenase	0.473	–
gb|ELV65774.1|	1,4-alpha-glucan branching enzyme	0.608	–
gb|ELV79446.1|	Glucose-1-phosphate adenylyltransferase	0.574	–
gb|EZQ53784.1|	N-acetylmannosamine-6-phosphate 2-epimerase	0.588	–
gb|EZQ45276.1|	Aldehyde dehydrogenase	0.592	1.772
gb|ELW32384.1|	ATP synthase F0, C subunit	1.789	–
gb|ELW24726.1|	ATP synthase F1, epsilon subunit	1.74	–
gb|EZQ54587.1|	Cytochrome C biogenesis protein ccma	0.402	–
gb|EZQ54591.1|	Cytochrome C biogenesis protein ccme	0.561	–
gb|ELV68907.1|	Cytochrome c-type biogenesis protein CcmF	–	0.511
**LIPID TRANSPORT AND METABOLISM**			
gb|EZQ53605.1|	Hydroxymyristoyl-ACP dehydratase, FadZ	0.482	–
gb|EZQ55626.1|	3-ketoacyl-CoA thiolase, FadI	0.599	1.623
gb|ELW32281.1|	Fatty oxidation complex, alpha subunit FadJ	0.626	1.554
gb|EZQ51542.1|	CDP-diacylglycerol pyrophosphatase	0.482	0.618
**AMINO ACID TRANSPORT AND METABOLISM**			
gb|EZQ55115.1|	Leucine ABC transporter substrate-binding protein	0.648	–
gb|EZQ55595.1|	Histidine ABC transporter substrate-binding protein hisj	2.247	1.781
gb|EZQ54204.1|	Arginine transporter ATP-binding subunit	–	1.711
gb|EZQ51932.1|	Cystine transporter subunit	–	1.907
gb|EZQ54744.1|	Glutamine ABC transporter substrate-bindnig protein	–	2.079
gb|EZQ52911.1|	Glycine/betaine ABC transporter substrate-binding protein	–	2.515
gb|EZQ51636.1|	Asparagine synthetase asna	0.609	–
gb|EZQ52125.1|	L-asparaginase II	0.373	–
gb|EZQ45758.1|	Imidazole glycerol phosphate synthase	0.64	–
gb|ELV71854.1|	Tryptophan biosynthesis protein trpCF	0.587	–
gb|EZQ50112.1|	Isopropylmalate isomerase	0.508	–
gb|EZQ54388.1|	Phospho-2-dehydro-3-deoxyheptonate aldolase	0.64	–
gb|EZQ51398.1|	Chorismate mutase	0.555	–
gb|ELW25484.1|	L-serine ammonia-lyase	0.367	–
gb|EZQ52704.1|	Threonine dehydratase	0.302	–
gb|EZQ50425.1|	Lysine decarboxylase LdcC	0.253	–
gb|ELW30974.1|	Peptidase E	0.506	–
gb|EZQ51284.1|	Peptidase T	0.484	–
gb|EZQ55313.1|	Xaa-Pro aminopeptidase	0.607	–
gb|EZQ51284.1|	Peptidase T	0.484	–
gb|EZQ55313.1|	Xaa-Pro aminopeptidase	0.607	–
**NUCLEOTIDE TRANSPORT AND METABOLISM**			
gb|EZQ48811.1|	Adenine phosphoribosyltransferase	1.669	1.575
gb|EZQ54639.1|	Dihydropyrimidine dehydrogenase	0.4	–
gb|EZQ48800.1|	5′-nucleotidase	1.613	1.527
**COENZYME TRANSPORT AND METABOLISM**			
gb|EZQ51655.1|	Molybdopterin-guanine dinucleotide biosynthesis protein MobA	0.423	0.594
gb|EZQ54715.1|	Molybdopterin biosynthesis protein B	0.616	–
gb|EZQ54280.1|	Dethiobiotin synthetase	0.519	1.626
gb|EZQ50136.1|	Dihydrofolate reductase	0.647	–
gb|ELV83997.1|	Dihydroneopterin triphosphate pyrophosphatase	0.659	–
gb|EZQ53652.1|	Aspartate decarboxylase	1.733	–
**TRANSCRIPTION AND TRANSLATION**			
gb|EZQ54982.1|	50S ribosomal protein L29	1.502	–
gb|EZQ53200.1|	50S ribosomal protein L34	7.874	1.626
gb|EZQ52752.1|	30S ribosomal protein S21	1.664	–
gb|EZQ54820.1|	Translation initiation factor IF-1	2.178	–
gb|EZQ52380.1|	DNA-directed RNA polymerase subunit omega	1.515	–
gb|EZQ45319.1|	ATP-dependent RNA helicase DbpA	0.607	0.661
gb|EZQ53798.1|	Ribosome hibernation promoting factor HPF	1.518	1.796
gb|EZQ50278.1|	DNA helicase	0.628	–
gb|ELW21116.1|	Bacterial regulatory helix-turn-helix, lysR	0.531	–
gb|EZQ55349.1|	Fis family transcriptional regulator	0.456	–
gb|EZQ48867.1|	NrdR family transcriptional regulator	1.525	1.605
gb|EZQ54772.1|	DeoR faimly transcriptional regulator	0.48	–
gb|EZQ51719.1|	AraC family transcriptional regulator	0.509	–
gb|ELW36180.1|	HTH-type transcriptional regulator iscR	1.852	–
**DNA REPLICATION AND REPAIR**			
gb|EZQ53883.1|	Uracil-DNA glycosylase	0.644	–
gb|ELW24896.1|	DNA-3-methyladenine glycosylase 1	0.457	–
gb|ELW33519.1|	Exodeoxyribonuclease I	0.664	–
gb|ELW34722.1|	Exodeoxyribonuclease V, beta subunit	0.642	–
gb|ELW28536.1|	Exodeoxyribonuclease 10	0.635	–
**MEMBRANE BIOSYNTHESIS AND TRANSPORT**			
gb|ELV85274.1|	Outer membrane protein W	0.414	–
gb|ELW34382.1|	Protein TolA	1.57	–
gb|ELW30265.1|	Large conductance mechanosensitive channel protein	1.61	–
gb|EZQ52814.1|	Colicin uptake protein TolR	1.565	–
gb|EZQ55714.1|	Cell division protein FtsI	2.71	–
**PROTEIN POST-TRANSLATIONAL MODIFICATION AND CHAPERONE**			
gb|EZQ55019.1|	Peptidylprolyl isomerase	1.786	–
gb|EZQ53211.1|	Heat shock protein IbpA	–	0.629
gb|ELV64283.1|	Disulfide interchange protein DsbA	1.739	–
gb|EZQ50339.1|	Methionine sulfoxide reductase A	0.62	–
**INORGANIC ION TRANSPORT**			
gb|EZQ55057.1|	Ferrous iron transporter B	1.553	–
gb|EZQ51582.1|	Ferritin	0.581	–
gb|EZQ52832.1|	Molybdate transporter	–	0.623
gb|ELW31127.1|	Nitrite extrusion protein 1	0.38	–
**SIGNAL TRANSDUCTION**			
gb|EZQ54151.1|	Carbon starvation protein A	–	1.502
gb|EZQ51547.1|	Universal stress protein UspD	1.582	–
gb|ELW36511.1|	Inner membrane protein YjiY	0.481	–
gb|ELW38901.1|	Sensor protein KdpD	0.608	0.629
**SECRETION**			
gb|EZQ53138.1|	Preprotein translocase subunit SecA	1.527	–
gb|EZQ54180.1|	Preprotein translocase subunit SecE	1.534	–
gb|EZQ53826.1|	Preprotein translocase subunit SecG	2.146	–
gb|EZQ53265.1|	Type III secretory protein EscJ	1.957	–
**CELL DEFENSE**			
gb|EZQ51533.1|	Superoxide dismutase	2.433	–
gb|EZQ55206.1|	Cytochrome C peroxidase	0.381	–
gb|EZQ54745.1|	DNA starvation/stationary phase protection protein Dps	0.523	–
gb|EZQ51542.1|	Undecaprenyl-diphosphatase OS	0.633	0.618

*SICs means sublethally injured cells; CK_L_ means control of live cells; CK_D_ means control of dead cells; Fold change >1.5 and < 0.67 and P < 0.05 means up- and down regulation; –means no significant fold change*.

## Discussion

### Carbohydrate, lipid, and amino acid transport and metabolism related DEP

In this study, proteins involved in glycolysis, tricarboxylic acid cycle (TCA), glyoxylate cycle, anaerobic respiration, disaccharide metabolism and other processes were down-regulated in SICs compared with CK_L_ (Table 1), indicating that carbohydrate metabolism in the SICs was inhibited. However, proteins involved in carbohydrate transport and ATP synthesis, including phosphotransferase system (PTS) sugar transporter, ATP synthase F0, C subunit and ATP synthase F1, epsilon subunit, were up-regulated in the SICs compared with CK_L_ (Table 1). The PTS is involved in both the transport and phosphorylation of a large number of carbohydrates, known as PTS carbohydrates, in movement toward these carbon sources by the phosphoenolpyruvate (PEP) sensor system (Postma et al., 1993). The up-regulation of PTS sugar transporter in the SICs could enhance the carbohydrate absorption capacity, resulting in the accumulation of carbohydrates in cells, which could be a protective agent for the SICs against HPCD stress. ATP synthase produces ATP from ADP in the presence of a proton gradient across the membrane (Guo et al., 2017). The up-regulation of ATP synthase in the SICs might be caused by the pH lowering effect of CO_2_ during HPCD treatment, helping establish a transmembrane difference of proton electrochemical potential that the ATP synthase could use to synthesize ATP (Khalimonchuk and Rödel, 2005). The up-regulation of ATP synthase in the SICs could intake extracellular proton and make a membrane balance of protons, thus protecting the SICs against HPCD stress. Compared with CK_D_, 5 carbohydrate metabolism related proteins were down-regulated in the SICs (Table 1). The down-regulation of these proteins indicated that the SICs were in a dormant-like state with a relatively low carbohydrate metabolism activity, which could improve the survival of these cells under HPCD stress. Isocitrate lyase (ICL) and PTS sugar transporter was up-regulated in the SICs compared with CK_D_ (Table 1). ICL catalyzes the reversible formation of succinate and glyoxylate from isocitrate, a key step of the glyoxylate cycle, which operates as an anaplerotic route for replenishing the oxaloacetate in TCA (Ornston and Ornston, 1969). The decarboxylation of oxaloacetate is the initial step of gluconeogenesis at the phosphoenolpyruvate–pyruvate–oxaloacetate node (Sauer and Eikmanns, 2005). The up-regulation of ICL might enhance the gluconeogenesis in the SICs compared with CK_D_, resulting in the increase of glucose content in the SICs. Meanwhile, the up-regulation of PTS sugar transporter could also increase the sugar contents in the SICs. These results indicated that sugar accumulation and low carbohydrate degradation activity might be an important protective strategy for the SICs against HPCD stress.

Proteins involved in lipid synthesis, lipid degradation and phospholipid metabolism, including hydroxymyristoyl-ACP dehydratase (FabZ), 3-ketoacyl-CoA thiolase (FadI), fatty oxidation complex, alpha subunit (FadJ) and CDP-diacylglycerol pyrophosphatase (CDAGP) were down-regulated in the SICs compared with CK_L_. FadJ and FadI were up-regulated, but CDAGP was down-regulated in the SICs compared with CK_D_ (Table 1). FabZ efficiently catalyzes the dehydration of short chain b-hydroxyacyl-ACPs and long chain saturated and unsaturated b-hydroxyacyl-ACPs in the dissociated, type II fatty acid biosynthesis (Heath and Rock, 1996). The down-regulation of FabZ in the SICs indicated that the lipid synthesis of the SICs might be inhibited. Both FadJ and FadI are involved in fatty acid beta-oxidation: FadJ catalyzes the formation of a hydroxyacyl-CoA by addition of water on enoyl-CoA, and FadI catalyzes the final step of fatty acid oxidation in which acetyl-CoA is released and the CoA ester of a fatty acid two carbons shorter is formed (Campbell et al., 2003). FadJ and FadI were down-regulated in the SICs when compared with CK_L_, but were up-regulated when compared with CK_D_, indicating the lipid degradation of the SICs was decreased by HPCD treatment. CDAGP synthesizes phosphatidate from CDP-diacylglycerol, which is part of phospholipid metabolism (Icho et al., 1985). The down-regulation of CDAGP in the SICs might result in less phospholipid synthesis. Overall, these results indicated that the lipid transport and metabolism in the SICs were decreased, and the low metabolic activity might improve the survival of these cells under HPCD stress.

Compared with CK_L_, proteins involved in amino acid transport, amino acid synthesis and amino degradation, except for histidine ABC transporter substrate-binding protein HisJ, were down-regulated in the SICs (Table 1), indicating that amino acid transport and metabolism in the SICs might be decreased. However, proteins involved in amino acid transport, including histidine ABC transporter substrate-binding protein HisJ, arginine transporter ATP-binding subunit, cystine transporter subunit, glutamine ABC transporter substrate-binding protein, glycine/betaine ABC transporter substrate-binding protein, were up-regulated in the SICs compared with CK_D_ (Table 1). ATP binding cassette (ABC) transporters is a large class of transporters that are responsible for the ATP powered translocation of many substrates across membranes, besides, ABC transporters mainly function as importers in prokaryotes, where they mediate the uptake of essential nutrients, such as amino acids, sugars, and essential metals (Rees et al., 2009). The up-regulation of these ABC transporters might result in more amino acids importing into the SICs. Amino acids could function as protective agent and repair material for the SICs (Bi et al., 2015). Therefore, the differences in the expression of these ABC transporters between the SICs and dead cells might contribute to the survival of the SICs under HPCD stress.

### Nucleotide, coenzyme transport, and metabolism related DEP

Adenine phosphoribosyltransferase (APT) and 5′-nucleotidase were up-regulated in the SICs compared with CK_L_ and CK_D_ (Table 1). APT catalyzes a reaction between adenine and 5-phosphoribosyl-1-pyrophosphate (PRPP) to produce adenosine monophosphate (AMP) and pyrophosphate, providing an alternative way of making AMP rather than synthesizing purine nucleotides *de novo* (Sin and Finch, 1972). The up-regulation of APT could provide AMP for the SICs in an energy-saving way. Dihydropyrimidine dehydrogenase (PRE) was down-regulated in the SICs compared with CK_L_ (Table 1). PRE is involved in pyrimidine base degradation, and it catalyzes physiologically the reduction of uracil to 5,6-dihydrouracil (DHU) and the reduction of thymine to 5,6-dihydrothymine (DHT) by using NADH as a specific cosubstrate (Hidese et al., 2011). The down-regulation of PRE could result in less pyrimidine base degradation in the SICs, which was in accordance with the dormant state of low biological activity of the SICs.

Proteins involved in Mo-molybdopterin guanine dinucleotide (Mo-MGD) cofactor biosynthesis, folic acid synthesis and biotin synthesis were down-regulated in the SICs compared with CK_L_ (Table 1). Molybdopterin-guanine dinucleotide biosynthesis protein (MobA) can transfer a GMP moiety from GTP to Mo-molybdopterin (Mo-MPT) cofactor (Moco or molybdenum cofactor) to form Mo-MGD cofactor and inorganpyrophosphate (Palmer et al., 1994). Molybdopterin biosynthesis protein B (MobB) is not required for the biosynthesis of Mo-MGD cofactor, and not necessary for the formation of active molybdoenzymes, but may act as an adapter protein to achieve the efficient biosynthesis and utilization of MGD (McLuskey et al., 2003). The down-regulation of MobA and MobB may decrease the transfer from GTP to GMP, resulting in less energy release, which was in accordance with the decreased energy demand of the SICs. Dethiobiotin synthetase (BioD) was down-regulated in the SICs when compared with CK_L_, but was up-regulated when compared with CK_D_ (Table 1). BioD can synthesize dethiobiotin from 7,8-diaminononanoate, which is part of the pathway biotin biosynthesis (Krell and Eisenberg, 1970). The down-regulation of BioD might decrease the biosynthesis of biotin, which may further reduce carbohydrate, lipid and protein metabolism since biotin was an important co-factor for carboxylases. These results indicated that the SICs might have a lower biotin synthesis capacity than the CK_L_, but a higher biotin synthesis capacity than the CK_D_. Dihydrofolate reductase (FolA) and dihydroneopterin triphosphate pyrophosphatase (NUDB) are key enzymes in folate metabolism: FolA synthesizes 5,6,7,8-tetrahydrofolate from 7,8-dihydrofolate (Iwakura et al., 2006); NUDB catalyzes the hydrolysis of dihydroneopterin triphosphate to dihydroneopterin monophosphate and pyrophosphate (Gabelli et al., 2007). The down-regulation of FolA and NUDB might decrease the biosynthesis of folate, which may further reduce amino acid and nucleotide synthesis since folate was an important co-factor for one carbon unit transferases.

### Transcription and translation, DNA replication, and repair related DEP

Proteins involved in rRNA binding or functioned as structural constituent of ribosome, including 50S ribosomal protein L29, 50S ribosomal protein L34 and 30S ribosomal protein S21, were up-regulated in the SICs compared with CK_L_. Ribosome hibernation promoting factor (HPF) was up-regulated while ATP-dependent RNA helicase (DbpA) was down-regulated in the SICs compared with CK_L_ and CK_D_ (Table 1). DbpA is involved in the assembly of the 50S ribosomal subunit, which has an RNA-dependent ATPase activity for 23S rRNA, and a 3′ to 5′ RNA helicase activity that uses the energy of ATP hydrolysis to destabilize and unwind short rRNA duplexes (Fuller-Pace et al., 1993). The down-regulation of DbpA indicated that the assembly of the 50S ribosomal submits might be decreased. As a negative regulation factor of translation, HPF can promote and stabilize dimerization of 70S ribosomes by the ribosome modulation factor (RMF), leading to the formation of inactive 100S ribosomes during stationary phase (Ueta et al., 2008). The up-regulation of HPF suggested that the ribosomes activity of the SICs might be inactive. These results showed that the translation activity of the SICs might be decreased although ribosomal proteins were up-regulated, which was in agreement with the dormant state with low biological activity of the SICs.

As shown in Table 1, transcription promoters including bacterial regulatory helix-turn-helix (LysR), Fis family transcriptional regulator (Fis), and AraC family transcriptional regulator (AraC) were down-regulated while transcription repressor including NrdR family transcriptional regulator (NrdR) and HTH-type transcriptional regulator (IscR) were up-regulated in the SICs compared with CK_L_. NrdR was also up-regulated in the SICs compared with CK_D_. LysR activates the transcription of the lysA gene encoding diaminopimelate decarboxylase and is also a negative regulator of its own expression (Stragier and Patte, 1983). Fis activates ribosomal RNA transcription, which plays a direct role in upstream activation of rRNA promoters, and can bind to a recombinational enhancer sequence required to stimulate hin-mediated DNA inversion (Ross et al., 1990). Fis can also prevent initiation of DNA replication from *oriC* to ensure the efficiency of transcription (Wold et al., 1996). AraC controls the expression of at least six genes involved in the transport and catabolism of L-arabinose and regulates initiation of transcription of the *araBAD* operon and its own synthesis (Soisson et al., 1997). The down-regulation of LysR, Fis, and AraC indicated that the transcription of the lysA gene, ribosomal RNA, and araBAD operon might be decreased. As transcriptional repressor, NrdR differentially regulates transcription of *nrdAB, nrdHIEF*, and *nrdD* genes encoding ribonucleotide reductase in aerobic growth by binding to NrdR boxes in the promoter regions to alter promoter activity (Torrents et al., 2007). IscR is a transcriptional repressor of the *iscRSUA* operon, which is involved in the assembly of Fe-S clusters into Fe-S proteins (Schwartz et al., 2001). The up-regulation of NrdR and IscR might cause the transcription of NrdR boxes and *iscRSUA* operon decreasing. These results indicated that the transcription activity of the SICs might be decreased. During transcription, the double chain of DNA was revealed and exposed to the environment, which may increase the damage to nucleic acid under stress conditions. The decrease in transcription activity of the SICs may result in a highly compressed helical DNA, which could avoid severe damage from HPCD stress and induce the cells entry into a dormant state to survive.

Proteins involved in DNA replication and repair were all down-regulated in the SICs compared with CK_L_ (Table 1), indicating the SICs had a lower DNA replication and repair activity. Similar results were found by others (Wu et al., 2012; Wang et al., 2016). Wang et al. (2016) found that all proteins in related to DNA replication and transcription in *S. aureus* cells were down-regulated after treatment with juglone for 2 h. Wu et al. (2012) found that four proteins involved in DNA replication of *Lactobacillus casei* were down-regulated to lactic acid treatment at pH 3.5. The decrease in DNA replication might have a protective effect on the nuclear region while the decrease in repair activity might induce more mutation, which might have a higher resistance to the HPCD stress and survive. Since the SICs induced by HPCD had a low capacity in DNA replication and repair, some technologies (e.g., UV; gamma irradiation; plasma) targeted to action on the DNA might easily kill the SICs, which may open up the possibility of combination HPCD and these technologies as hurdle approaches for food preservation.

### Membrane biosynthesis and inorganic ion transport related DEP

Proteins involved in membrane biosynthesis, except for outer membrane protein W (OmpW), were up-regulated in the SICs compared with CK_L_ (Table 1). Large conductance mechanosensitive channel protein (MSCL) forms a nonselective ion channel with a conductance of about 4 nanosiemens, which will open in response to a pressure causing sublethal injury in the membrane, to avoid cell disruption and death (Sukharev et al., 1994). The up-regulation of MSCL indicated that the SICs had a pressure response to HPCD treatment. Colicin uptake protein TolR (TolR) and protein TolA (TolA) are involved in the TonB-independent uptake of group A colicins (colicins A, E1, E2, E3, and K) (Kampfenkel and Braun, 1993). The up-regulation of TolR and TolA may increase the transport of group A colicins in The SICs. Cell division protein FtsI (FtsI) is involved in the pathway peptidoglycan biosynthesis, which catalyzes cross-linking of the peptidoglycan cell wall at the division septum (Ishino and Matsuhashi, 1981). The up-regulation of FtsI in the SICs could increase the cross-linking of peptidoglycan, which might be a response to the high pressure under HPCD treatment.

Ferritin (FTNA) and nitrite extrusion protein 1 (NarK) was down-regulated in the SICs compared with CK_L_ while molybdate transporter was down-regulated in the SICs compared with CK_D_ (Table 1). The down-regulation of these proteins might decrease the transport of some inorganic ion such as iron, molybdate, and nitrite. However, ferrous iron transporter B (FEOB) was up-regulated in the SICs compared with CK_L_. FEOB is a transporter of a GTP-driven Fe^2+^ uptake system and a stimulus of cellular response to DNA damage (Marlovits et al., 2002). The up-regulation of FEOB might increase the intake of Fe^2+^ in the SICs or be a response to DNA damage under HPCD stress.

### DEP involved in other processes

Among the DEP involved in protein post-translational modification and chaperone, heat shock protein IbpA was down-regulated in the SICs compared with CK_D_, while disulfide interchange protein (DsbA) was up-regulated in the SICs compared with CK_L_ (Table 1). IbpA is associated with aggregated proteins, together with IbpB, to stabilize and protect them from irreversible denaturation and extensive proteolysis during heat shock and oxidative stress (Kuczynska-Wiśnik et al., 2002). The down-regulation of IbpA in the SICs under HPCD stress might be caused by the low temperature and anaerobic environment during HPCD treatment, which would not trigger the IbpA expression. DsbA is required for disulfide bond formation in some periplasmic proteins such as PhoA or OmpA and actes by transferring its disulfide bond to other proteins and itself is reduced in the process (Kishigami et al., 1995). The up-regulation of DsbA might increase the formation of disulfide bond in outer membrane protein, which could improve the stability of outer membrane protein and thus contribute to survival of the SICs under HPCD stress.

Among the DEP involved in signal transduction, carbon starvation protein A (CSTA) and universal stress protein UspD (USPD) were up-regulated in the SICs compared with CK_D_ and CK_L_, respectively (Table 1). CSTA can utilize peptide during carbon starvation, which is a stress response of cell to starvation (Schultz and Matin, 1991). The up-regulation of CSTA might increase the breaking down of peptide to amino acids, which could act as cell protection agent under stress. USPD is required for resistance to DNA-damaging agents and its expression can be induced by starvation, heat shock and toxicants (Bochkareva et al., 2002). The up-regulation of USPD could improve the resistance of the SICs to DNA damage by HPCD treatment.

Among the DEP involved in cell defense, superoxide dismutase (SOD) was up-regulated while cytochrome C peroxidase (YHJA) and DNA starvation/stationary phase protection protein (DPS) was down-regulated in the SICs compared with CK_L_ (Table 1). SOD can defend against the toxicity of superoxide by scavenge it with hydrogen ion to form oxygen and hydrogen peroxide (Imlay and Imlay, 1996). YHJA can catalyze hydrogen peroxide and ferrocytochrome c to form water and ferricytochrome c (Partridge et al., 2007). The up-regulation of SOD and down-regulation of YHJA indicated that the SICs had an increased SOD radical destroying capacity but a decreased YHJA radical destroying capacity. DPS can bind the chromosome non-specifically, forming a highly ordered and stable dps-DNA co-crystal within which chromosomal DNA is condensed and protected from diverse damages, including oxidative damage, UV and gamma irradiation, iron and copper toxicity, thermal stress and acid and base shocks (Nair and Finkel, 2004). The down-regulation of DPS indicated that the SICs had a lower protection capacity to DNA damage, which was in agreement with that the SICs had a lower DNA repair capacity. The sublethal injury in DNA could result in some mutations, which might have a higher resistance to the HPCD stress and survive.

Proteins involved in secretion were all up-regulated in the SICs compared with CK_L_ (Table 1), indicating that the SICs might have a higher protein secretion capacity. However, the physiological function of the increased secretion was unknown, which needed further study to confirm.

## Conclusion

We explored the molecular basis involved in the formation of sublethally injured *E. coli* O157:H7 induced by HPCD using iTRAQ proteomic methods. The changes in protein expression might be associated with pathways contributing to the formation of the SICs and their survival. Firstly, the SICs showed stress response to HPCD, with an increase of carbohydrate transport, peptidoglycan synthesis and disulfide bond formation, which could function as a barrier protecting the cells against environmental stresses. Secondly, the SICs showed DNA damage response to HPCD, with a decreased DNA repair capacity, which might generate more mutations with a higher resistance to HPCD and survive. Thirdly, activities of main metabolic processes as carbohydrate and nucleotide decomposing, transport and metabolism of lipid, amino acid and coenzyme were decreased, and transcription, translation and DNA replication were repressed during formation of The SICs. In conclusion, decreased metabolic activity, repressed cell division and enhanced survival ability in *E. coli* O157:H7 might cause the formation of the SICs under HPCD.

## Author contributions

XB: Carrying out the experiments and writing the manuscript; YW: Giving advice and assistance for the experiment; XH: Reviewing the manuscript and giving advice; XL: Designing the experiment and reviewing the manuscript.

### Conflict of interest statement

The authors declare that the research was conducted in the absence of any commercial or financial relationships that could be construed as a potential conflict of interest.
