# Supplementary material for: iTRAQ-Based Proteomic Analysis of Sublethally Injured Escherichia coli O157:H7 Cells Induced by High Pressure Carbon Dioxide
Source: Front Microbiol. 2017 Dec 18;8:2544. doi: 10.3389/fmicb.2017.02544 (PMC5770692; doi:10.3389/fmicb.2017.02544)
Supplement: Supplementary file 1 [file Table1.DOCX]

**Table S1** Summary of protein identification for sublethally injured, live and dead *Escherichia coli* O157:H7 cells using the iTRAQ platform.

| Category | Identified number |
| --- | --- |
| Total spectra | 342823 |
| Spectra | 62602 |
| Unique spectra | 60678 |
| Peptide | 17322 |
| Unique peptide | 17060 |
| Protein | 2446 |
